# Recurrent loss of HMGCS2 shows that ketogenesis is not essential for the evolution of large mammalian brains

**DOI:** 10.7554/eLife.38906

**Published:** 2018-10-16

**Authors:** David Jebb, Michael Hiller

**Affiliations:** 1 Max Planck Institute of Molecular Cell Biology and GeneticsDresdenGermany; 2 Max Planck Institute for the Physics of Complex SystemsDresdenGermany; 3 Center for Systems Biology DresdenDresdenGermany

**Keywords:** mammals, cetaceans, fruit bats, elephants, Other

## Abstract

Apart from glucose, fatty acid-derived ketone bodies provide metabolic energy for the brain during fasting and neonatal development. We investigated the evolution of *HMGCS2*, the key enzyme required for ketone body biosynthesis (ketogenesis). Unexpectedly, we found that three mammalian lineages, comprising cetaceans (dolphins and whales), elephants and mastodons, and Old World fruit bats have lost this gene. Remarkably, many of these species have exceptionally large brains and signs of intelligent behavior. While fruit bats are sensitive to starvation, cetaceans and elephants can still withstand periods of fasting. This suggests that alternative strategies to fuel large brains during fasting evolved repeatedly and reveals flexibility in mammalian energy metabolism. Furthermore, we show that *HMGCS2* loss preceded brain size expansion in toothed whales and elephants. Thus, while ketogenesis was likely important for brain size expansion in modern humans, ketogenesis is not a universal precondition for the evolution of large mammalian brains.

## eLife digest

Our brain requires a lot of energy to work properly. Sugars are usually the main type of fuel for the body, but when they run low – for example during a food shortage – fat, in the form of fatty acids, can be used instead. However, the brain cannot directly process these molecules; instead, fatty acids need to go through ketogenesis, a process that turns fat into ketone bodies, which the organ can then burn. Scientists believe that the ability to create ketone bodies was essential for us to evolve large brains. Yet, it is still unclear if all mammals can transform fatty acids into ketone bodies. One way to look into this question is to track whether other species have HMGCS2, the main enzyme that drives ketogenesis.

Jebb and Hiller examined the genomes of 70 different species of mammals for the gene that codes for HMGCS2. The comparisons revealed that cetaceans (whales, dolphins and porpoises), Old World fruit bats and the African savanna elephant have all independently lost their working version of HMGCS2. Yet, many members of these three groups have evolved brains that are large for their body size. The genetic analyses showed that dolphins and elephants developed big brains after the enzyme became inactive, challenging the idea that HMGCS2 – and by extension ketogenesis – is always required for the evolution of large brains.

These results may also be useful for conservation efforts. Many fruit bats across the world are severely threatened, and their lack of ketogenesis could explain why these animals are highly sensitive to starvation and quickly die when food becomes scarce.

## Introduction

Periods of fasting are a common event for many animals (Secor and Carey, 2016). Fasting occurs due to natural food scarcity or as part of the life history strategy, for example during hibernation or migration. During fasting, the organism relies on stored sources of energy such as glucose in the form of glycogen and fatty acids (Secor and Carey, 2016). In addition, ketone bodies become an alternative fuel source that is important for many mammals to survive episodes of fasting or starvation (Baird et al., 1972; Bouchat et al., 1981; Sicart et al., 1978). For example, ketone bodies are used as an energy source in hibernating ground squirrels or elephant seal pups during their post-weaning fasting period (Krilowicz, 1985; Castellini and Costa, 1990). Notably, while the brain cannot metabolize fatty acids, ketone bodies can cross the blood-brain barrier and provide fuel under conditions of low blood glucose levels. For example, after starving for 3 days, the human brain takes 25% of its energy from ketone bodies and if fasting continues, ketone bodies replace glucose as the predominant fuel for brain metabolism (Owen et al., 1967; Hasselbalch et al., 1994). During the neonatal period, the developing human brain has high energy requirements and also relies on ketone bodies as a major fuel (Cunnane and Crawford, 2003; Cahill, 2006). Given their importance in fueling large, energetically expensive brains, it has been posited that ketone bodies do not only have an important role during fasting, but have also been crucial for brain expansion during human evolution (Cunnane and Crawford, 2003; Wang et al., 2014).

**Figure 1.**
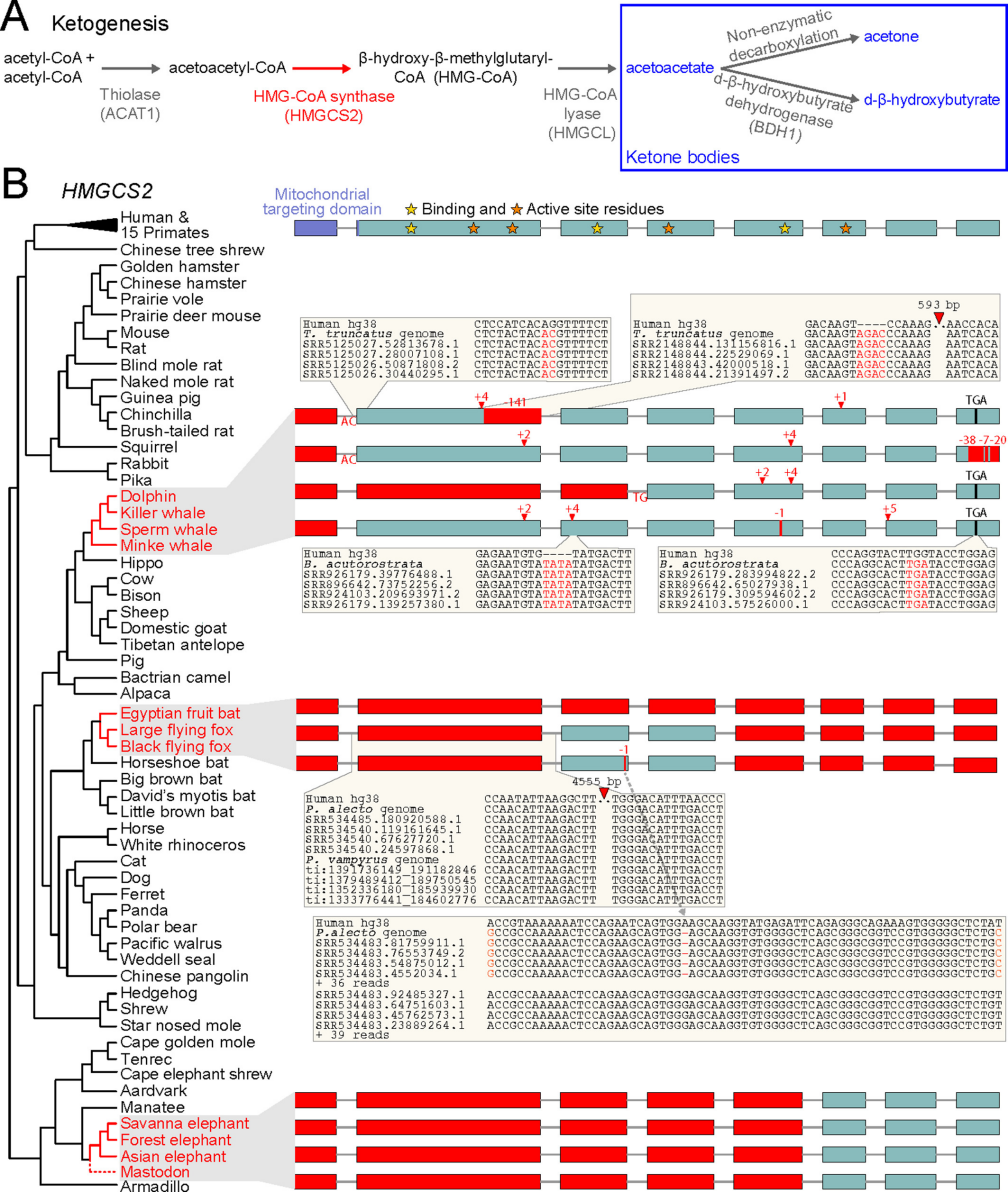
Evolution of ketogenesis in placental mammals. (**A**) Biosynthesis of ketone bodies (blue font). With the exception of the mitochondrial HMG-CoA synthase (HMGCS2, red font), the other two enzymes required for acetoacetate production also have roles in amino acid metabolism and are thus pleiotropic. BDH1 is only required for converting acetoacetate into d-β-hydroxybutyrate. (**B**) Recurrent loss of *HMGCS2* in three independent lineages (red font). All species in black font have an intact *HMGCS2* reading frame. Boxes are coding exons proportional to their size, introns are shown as horizontal lines. Red boxes are exon deletions. In-frame stop codon, frameshifting insertion/deletion and splice site-disrupting mutations are indicated. With the exception of the heterozygous 1 bp deletion in the black flying fox that has a read support of ~50:50 for the derived and ancestral allele and reveals two distinct haplotypes (inset), all shown mutations are supported by at least 30 reads with no support for the ancestral allele (Supplementary file 1). Insets exemplify the validation of inactivating mutations by showing the local genomic context and four reads.

Ketone bodies comprise acetoacetate, acetone, and d-β-hydroxybutyrate (Figure 1A) and are mainly produced in the liver by ketogenesis. This metabolic process occurs in the mitochondria and uses fatty acid-derived acetyl-CoA to generate the water-soluble, acidic ketone bodies, which are secreted into the blood. The rate limiting step of ketogenesis is the production of 3-hydroxy-3-methylglutaryl-CoA (HMG-CoA) by HMG-CoA synthase (Hegardt, 1999). Mammals possess two HMG-CoA synthases that originated by gene duplication. While the cytosolic enzyme, encoded by *HMGCS1*, is broadly expressed and is necessary to produce cholesterol (Hegardt, 1999), the mitochondrial HMG-CoA synthase, encoded by *HMGCS2*, is primarily expressed in the liver and is only used for ketone body production. *HMGCS2* is required for ketogenesis, as mutations in the human gene and mouse gene-knockdown experiments abolish or greatly reduce ketogenesis (Bouchard et al., 2001; Ramos et al., 2013; Thompson et al., 1997; Wolf et al., 2003; Pitt et al., 2015; Cotter et al., 2014). HMG-CoA synthase-2 deficiency in human can lead to coma after fasting for more than 22 hours due to low glucose levels (Thompson et al., 1997; Morris et al., 1998). Human individuals with *HMGCS2* mutations therefore require regular carbohydrate intake but show no other symptoms, suggesting that this deficiency is probably underdiagnosed.

**Figure 1—figure supplement 1.**
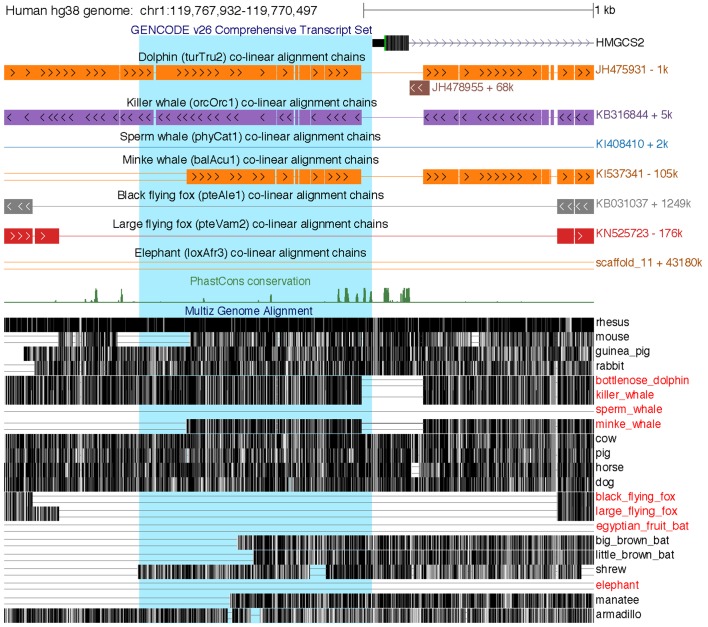
Loss of the *HMGCS2* promoter region in the sperm whale, pteropodids, and the elephant. The figure shows the 2.5 kb genomic region around *HMGCS2* exon 1 in the UCSC human (hg38 assembly) genome browser (Casper et al., 2018). The 1 kb region upstream of the transcription start site, highlighted in blue background, is deleted in the sperm whale and also lost in fruit bats and elephant, as shown by the co-linear pairwise alignment chains (blocks are aligning regions, a single line represents the shared deletion, double lines represent regions that do not align between the human and the query genome).

**Figure 1—figure supplement 2.**
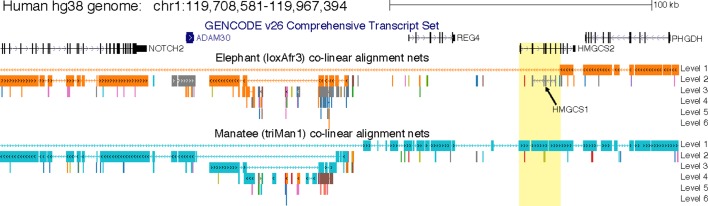
Deletion of a large part of *HMGCS2* in elephant. Screenshot of the human UCSC genome browser shows the locus of the *NOTCH2*, *ADAM30*, *REG4*, *HMGCS2* and *PHGDH* genes. Co-linear pairwise alignment chains to the African savanna elephant (loxAfr3 assembly) and the manatee (presumably its closest sister species [Poulakakis and Stamatakis, 2010]) show that several rearrangements, including probably two nested inversions, happened in this locus. Importantly, while *NOTCH2*, *ADAM30* and *PHGDH* align to both species, the elephant exhibits a large deletion that includes *REG4* and most of *HMGCS2* (yellow background, see Figure 1B). Alignment chains to the African savanna elephant loxAfr4 assembly are virtually identical and confirm the partial *HMGCS2* deletion. The grey elephant chain (arrow) that only aligns exons but not introns of *HMGCS2* is the elephant *HMGCS1* gene. As described in the main text, searching unassembled reads of 2 individuals of the African savanna elephant (whose genome has been sequenced) and read data of the African forest elephant, the Asian elephant and the mastodon did not reveal any evidence for the presence of the deleted *HMGCS2* exons or the *REG4* gene, suggesting that this genomic region was already deleted in ancestor of elephants and mastodons.

**Figure 1—figure supplement 3.**
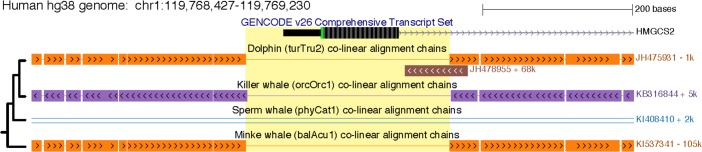
A shared ~ 270 bp deletion removed *HMGCS2* exon one in cetaceans. The figure shows exon one in the UCSC human (hg38 assembly) genome browser. This exon is deleted in all cetaceans (yellow background) and has the same breakpoints, as shown by the co-linear pairwise alignment chains (blocks are aligning regions, a single line represents the shared deletion). This region was subsequently removed by another large deletion in the sperm whale lineage. The phylogenetic relationships of the four species are indicated on the left side.

**Figure 1—figure supplement 4.**
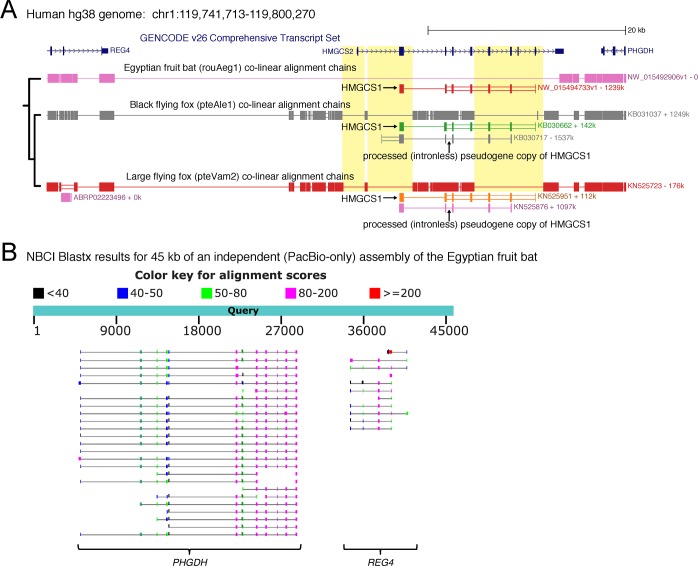
Large deletions in the *HMGCS2* locus in pteropodid fruit bats. (**A**) Screenshot of the human UCSC genome browser shows the *HMGCS2* locus with its flanking genes and co-linear pairwise alignment chains to three pteropodid bats. Chains that align the paralog *HMGCS1* or a processed *HMGCS1* pseudogene in flying foxes are labeled. Please note that we show in this figure the transcript ENST00000369406, which includes an alternative exon three that is skipped in the transcript shown in Figure 1B (ENST00000544913). Yellow background highlights the deletion of exon 1, a separate deletion that removed exon 2 (shown in detail in the inset in Figure 1B) and a deletion that removed four consecutive exons downstream. At least the second deletion exhibits shared breakpoints between both flying foxes, showing that loss of *HMGCS2* already occurred in the ancestor of both species. The phylogenetic relationships of the three species are indicated on the left side. (**B**) To validate the deletion of *HMGCS2* in the Egyptian fruit bat, we used an independent PacBio assembly. The locus around the neighboring *PHGDH* and *REG4* gene is covered by several large PacBio reads. Blastx results only reveal the presence of *PHGDH* and *REG4* but no evidence of *HMGCS2*. Consistent with *HMGCS2* deletion, the *PHGDH* and *REG4* are separated by ~ 50 kb in human but only ~ 6 kb in the Egyptian fruit bat.

**Figure 1—figure supplement 5.**
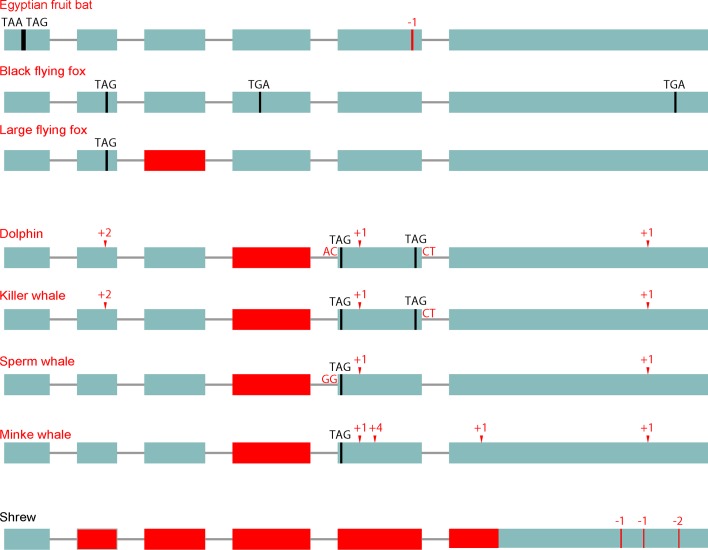
Loss of the d-β-hydroxybutyrate producing *BDH1* gene. BDH1 converts the ketone body acetoacetate into the ketone body d-β-hydroxybutyrate (Figure 1A). *BDH1* is a non-essential gene as a knockout in mouse results in the absence of d-β-hydroxybutyrate and a compensating increase in acetoacetate without major problems (Otsuka et al., 2017). Furthermore, Bdh1 knockout mice survive a 48 hr fast. We found that pteropodids and cetaceans but not the elephant exhibit several gene-inactivating mutations in this gene (visualization as in Figure 1B). RELAX analysis shows that *BDH1* also evolved under relaxed selection in pteropodids and cetaceans (Supplementary file 2). In addition, we found that this gene has several inactivating mutations and a large deletion in the shrew. Importantly, *ACAT1*, *HMGCS2* and *HMGCL* are intact in the shrew, suggesting that acetoacetate and acetone are the primary ketone bodies in this species. None of the other analyzed placental mammals had inactivating mutations in *BDH1*.

Here we investigated the evolution of *HMGCS2* in mammals. Unexpectedly, we identified three independent losses of this gene in cetaceans (dolphins and whales), pteropodids (Old World fruit-eating bats) and Elephantimorpha (elephants and mastodons). Remarkably, these species have relatively large brains, suggesting that, unlike in humans, ketone bodies are not strictly required for fueling complex brains. Furthermore, we show that in the cetacean and Elephantimorpha clades *HMGCS2* was lost before brain size expansion happened, suggesting that the lack of ketogenesis did not prohibit the evolution of large brains in these lineages. While strong conservation of *HMGCS2* in other mammals indicates that ketogenesis is a crucial metabolic process, the recurrent loss of this gene highlights an unexpected flexibility in mammalian energy metabolism.

## Results and discussion

To investigate the evolution of *HMGCS2*, we used a previously published whole genome alignment to inspect the gene sequence and the surrounding locus across 70 placental mammals (Sharma and Hiller, 2017). Surprisingly, we discovered that three independent lineages (cetaceans, pteropodids and the African savanna elephant) exhibit large deletions that remove *HMGCS2* exons or gene-inactivating mutations that shift the *HMGCS2* reading frame and destroy conserved splice site dinucleotides (Figure 1B). All three lineages have a deletion of exon one that encodes the mitochondrial targeting domain; such a deletion causes HMG-CoA synthase-2 deficiency in human individuals (Pitt et al., 2015). Other mutations affect exons encoding key residues required for HMG-CoA synthase catalytic activity and leave little of the coding sequence intact. Together with the deletion of the promoter region in pteropodids, the elephant and the sperm whale (Figure 1—figure supplement 1), this shows that three mammalian lineages lost the enzyme that is required for ketogenesis.

In cetaceans and pteropodids, the remnants of the once-functional *HMGCS2* gene are located in a conserved genomic context with *REG4* upstream and *PHGDH* downstream. In elephant, the three remaining *HMGCS2* exons also occur in the same genomic locus adjacent to the conserved *PHGDH* gene, but inversions that already happened in the ancestor of elephants and the closely related manatees rearranged the locus upstream of *HMGCS2* (Figure 1—figure supplement 2). These rearrangements were succeeded by a large deletion in the elephant lineage that removed the first five *HMGCS2* exons together with the *REG4* gene.

To rule out that the gene-inactivating mutations are sequencing or genome assembly errors, we validated all smaller mutations and exon deletions with unassembled sequencing reads from the SRA and TRACE archives using blastn. All 22 mutations in cetaceans were confirmed by at least 30 reads, with no support for the non-gene-inactivating allele (Figure 1B, Supplementary file 1). This includes the deletion of exon one that exhibits shared breakpoints in the toothed and baleen whale lineages (Figure 1—figure supplement 3), which strongly suggests that this deletion and thus *HMGCS2* loss already occurred before the split of the main cetacean lineages (Figure 1C). This is further supported by the 2 bp frameshifting insertion in exon two that is shared between killer whale and minke whale, and was later deleted in dolphin and sperm whale.

In pteropodid bats, the ~4.5 kb deletion that removed coding exon two is validated by sequencing reads and is shared between both flying foxes (Figure 1B), suggesting that *HMGCS2* was already lost in their common ancestor. Using the *HMGCS2* sequence of the David’s myotis bat, we detected no evidence for the presence of the deleted *HMGCS2* exons in unassembled sequencing reads of both flying fox species, while we readily found all exons of the *HMGCS1* paralog, showing that the search is sufficiently sensitive. In the Egyptian fruit bat, *HMGCS2* is entirely removed by a large deletion between the *REG4* and *PHGDH* genes, which we validated with an independent PacBio assembly (Figure 1—figure supplement 4). Consistent with ongoing gene erosion, the 1 bp deletion in exon three is heterozygous in the black flying fox (Figure 1B).

To rule out that the partial gene deletion in the African savanna elephant is an assembly error, we used the manatee *HMGCS2* sequence. Sensitive blastn searches found no significant hits for the deleted *HMGCS2* exons or the deleted neighboring *REG4* gene in the unassembled sequencing reads of two different savanna elephant individuals (Cortez et al., 2014). In contrast, the three remaining *HMGCS2* exons as well as all exons of the paralogous *HMGCS1* could be recovered. We also investigated related elephant species, making use of recently published sequence data from the African forest elephant and the Asian elephant (Palkopoulou et al., 2018; Reddy et al., 2015). Further, we queried sequence data from two American mastodons, extinct Elephantimorpha that split from elephants 28–24 Mya (Rohland et al., 2007). As for the savanna elephant, the three remaining *HMGCS2* exons and entire *HMGCS1* gene were found in all three species, while the deleted *HMGCS2* exons and the *REG4* gene were not found (Figure 1B). Parsimony suggests that the deletion, which removed large parts of *HMGCS2*, occurred prior to the divergence of mastodons and the elephant species.

We further found that the remaining *HMGCS2* sequence evolves under relaxed selection in cetaceans, pteropodids and Elephantimorpha (p<3e-3, Supplementary file 2). Together with the conserved genomic context, the lack of any evidence of a remaining functional *HMGCS2* in unassembled reads and the validated gene-inactivating mutations, we conclude that the main ketogenesis enzyme is lost in three independent mammalian lineages. Finally, we considered the possibility that HMGCS1, the cytosolic HMG-CoA synthase, may compensate for *HMGCS2* loss, which would require HMGCS1 to be localized in the mitochondria, where ketogenesis happens in other species. We found that the HMGCS1 protein of cetaceans, pteropodids and elephant does not possess a mitochondrial targeting domain. Furthermore, an analysis of available liver RNA-seq data from the minke whale and Egyptian fruit bat provides no indication of alternative or novel exons in *HMGCS1* that could encode such a targeting signal. Thus, HMGCS1 does not seem to be capable of compensating for the loss of *HMGCS2*, suggesting that ketogenesis is lost in cetaceans, pteropodids and Elephantimorpha.

Next, we investigated whether the loss of *HMGCS2* is associated with the loss of other enzymes in the ketogenesis pathway (Figure 1A). *ACAT1* and *HMGCL* do not exhibit inactivating mutations in cetaceans, pteropodids and the elephant, likely because the respective enzymes are not only required for the production of ketone bodies but are also involved in leucine and isoleucine metabolism. In contrast to these two pleiotropic genes, *BDH1* is only involved in converting acetoacetate into the ketone body d-β-hydroxybutyrate (Figure 1A). We found that *BDH1* exhibits several inactivating mutations and evolved under relaxed selection in cetaceans and pteropodids (Figure 1—figure supplement 5, Supplementary file 2). Overall, this suggests that the loss of *HMGCS2* is only associated with the loss of non-pleiotropic genes in the ketogenesis pathway.

The 59 other mammals, for which the genome assembly fully covered the *HMGCS2* locus (Figure 1B), do not exhibit inactivating mutations in this gene. Consistent with the presence of a functional gene, we further estimated an average non-synonymous/synonymous (dN/dS) ratio of 0.16, which indicates that *HMGCS2* evolves under strong purifying selection in other mammals.

**Figure 2.**
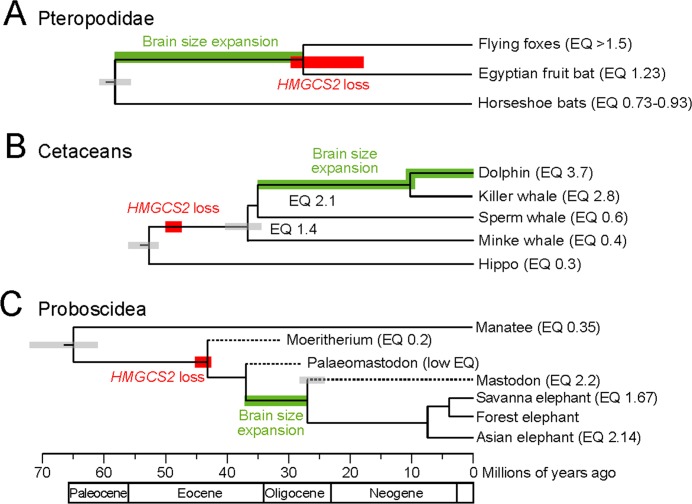
*HMGCS2* loss and brain size evolution. (**A**) In pteropodids, molecular dating estimates that the loss of *HMGCS2* happened 29–18 Mya and thus may overlap the split of the flying foxes and the Egyptian fruit bat. It is not possible to resolve whether gene loss happened before or after the split as *HMGCS2* is completely deleted in the Egyptian fruit bat. While horseshoe bats and other insectivorous bat lineages have brains not larger than expected for their body size (encephalization quotient (EQ) <1), brain size has increased in the lineage leading to the fruit bats that have EQ values > 1 (Stephan et al., 1981). Thus, brain size expansion presumably predates the loss of ketogenesis. (**B**) *HMGCS2* was already lost in the cetacean ancestor before the split of toothed and baleen whales ~ 36 Mya, as inferred from shared inactivating mutations in exons 1, 2 and 8. Molecular dating further estimates that the loss of this gene happened early on the cetacean branch 50–47 Mya. The cetacean ancestor had a brain slightly larger than expected for its body size with an EQ of 1.4. While EQ values increased and decreased in several cetacean lineages, brain size has greatly expanded in dolphins, reaching an EQ of 3.7 (Montgomery et al., 2013). Thus, brain size expansion in dolphins occurred after the loss of ketogenesis. (**C**) Early proboscids such as *Moeritherium*, an extinct lineage that split from other proboscids ~ 43 Mya, had brains about 20% of the size expected for a mammal of the same body size, and thus an EQ of 0.2 (Shoshani et al., 2006). Exact EQ values of Palaeomastodons are not known; however, fossils have a small braincase, which indicates a low EQ (Sanders et al., 2010; Benoit, 2015). In contrast, mastodons that diverged from elephants ~ 27 Mya had brains about twice as large as expected from their body size (EQ 2.2), similar to extant elephants (Shoshani et al., 2006). This suggests that brain size expansion happened in a period between 37 and 27 Mya. Molecular dating indicates that *HMGCS2* loss happened between 45 and 42 Mya, suggesting that the loss of ketogenesis precedes brain size expansion in the elephant lineage. Divergence times of extinct proboscid lineages were taken from (Shoshani and Tassy, 2013) and (Rohland et al., 2007). Supporting Information. 10.7554/eLife.38906.010Figure 2—source data 1.Sequence alignment.This file contains the *HMGCS2* sequence alignment (fasta format) including the sequences of the African forest elephant, the Asian elephant and the American mastodon. This alignment was used to date the loss of *HMGCS2*.

The observation that *HMGCS2* is well-conserved in the majority of mammals is consistent with ketogenesis being an important metabolic process. However, the recurrent loss of *HMGCS2* raises the question of which energy source is used by the brain during fasting. Consistent with the loss of ketogenesis in cetaceans, bottlenose dolphins do not produce ketone bodies after fasting for 3 days but are nevertheless able to maintain high blood glucose levels over this entire period (Ridgway, 2013). It was suggested that dolphins maintain high glucose levels by synthesis of glucose from non-carbohydrates (gluconeogenesis), in particular from glucogenic amino acids that are abundant in their diet (Ridgway, 2013). This suggests that ketogenesis became dispensable in dolphins and that *HMGCS2* was lost as a consequence of relaxed or no selection to maintain this gene. Similarly, the loss of ketogenesis in pteropodid fruit bats may be a consequence of the relatively constant availability of fruit year-round, which provides large quantities of glucose. This is in agreement with molecular dating, which estimates that the loss of *HMGCS2* happened rather late in the lineage leading to the fruit bat clade and may even have occurred independently after the split of the frugivorous flying foxes and the Egyptian fruit bat (Figure 2A). Consistent with lack of ketone bodies as alternative fuel, Egyptian fruit bats that were fasted for more than 24 hours in captivity frequently died (van der Westhuyzen, 1978). Thus, like HMG-CoA synthase-2 deficient human individuals, these bats are sensitive to starvation. Hence, while ketogenesis may have been lost under ancestral conditions of constantly available, glucose-rich food, the loss of *HMGCS2* may now represent a disadvantage, which will be of interest to ongoing conservation efforts for ecologically and economically important species in the pteropodid family. In contrast to cetaceans and fruit bats, little is known about how elephants respond to fasting; however, the following observation is consistent with the loss of ketogenesis. During musth, when elephant males experience longer periods of fasting and can lose 10% of their body weight, their blood becomes slightly more alkaline (Rasmussen and Perrin, 1999). This is contrary to an increased blood acidity that would be expected from an increasing production of acidic ketone bodies.

Given the importance of ketogenesis to provide energy to the brain during starvation, it is noteworthy that species in all three *HMGCS2*-loss lineages generally have large relative brain sizes (Stephan et al., 1981; Boddy et al., 2012). For example, the encephalization quotient (EQ), measuring the ratio between the observed brain size and the size expected for a mammal of the same body weight, is 3.7 for the bottlenose dolphin (Montgomery et al., 2013). Compared to human, dolphins and elephants are also among the few mammals that have a higher degree of neocortex folding, a measure that positively correlates with neuron number (Manger et al., 2012; Lewitus et al., 2014). Furthermore, while powered flight imposes a constraint on body and brain size in bats, pteropodid fruit bats exhibit a well-developed visual brain system and have brains nearly twice as large as that of insectivorous vesper bats of equal body weight (Stephan et al., 1981). Species in all three lineages also exhibit cognitive behaviors that are regarded as a sign of intelligence, exemplified by vocal learning and, in dolphins and elephants, by complex social structures, tool use and self-recognition (Krützen et al., 2005; Foerder et al., 2011; Poole et al., 2005; Prat et al., 2015; Plotnik et al., 2006). Thus, the loss of *HMGCS2* in independent large-brained species suggests that ketone bodies are not strictly required to fuel large mammalian brains during fasting.

Finally, the timing of *HMGCS2* loss has implications for understanding the general preconditions for brain size expansion during the evolution of mammals. While the loss of *HMGCS2* in pteropodids likely happened after brain size expansion in this lineage (Figure 2A), shared inactivating mutations show that *HMGCS2* was already inactivated in the cetacean ancestor, and thus prior to a period of brain size expansion that resulted in the large brains of dolphins (Boddy et al., 2012; Montgomery et al., 2013) (Figure 2B). For the elephant lineage, we used molecular dating to estimate that *HMGCS2* was lost around 45–42 Mya (Supplementary file 3). Thus, like in toothed whales, the loss of this gene likely occurred prior to the period that led to large relative brain sizes in modern elephants (Shoshani et al., 2006) (Figure 2C). Consequently, while ketogenesis was likely a crucial factor for brain size increase in humans (Cunnane and Crawford, 2003; Wang et al., 2014), the loss of ketogenesis has not prohibited drastic evolutionary brain size expansion in two other mammalian lineages.

In conclusion, we have identified three independent losses of *HMGCS2* in placental mammals. While this may contribute to starvation sensitivity in fruit bats, cetaceans and elephants can withstand periods of fasting. Hence, alternative strategies to fuel large brains during fasting have evolved at least twice, revealing flexibility in the energy metabolism of mammals. Finally, the timing of *HMGCS2* loss indicates that ketogenesis is not a universal precondition for the evolution of large mammalian brains. More generally, our results further highlight the potential of comparative gene analyses (Emerling and Springer, 2014; Meredith et al., 2009; Castro et al., 2014; Albalat and Cañestro, 2016; Lopes-Marques et al., 2017; Hecker et al., 2017; Gaudry et al., 2017; Sharma et al., 2018a; Sharma et al., 2018b; Meyer et al., 2018; Emerling et al., 2018) to reveal novel insights into the evolution of metabolic, physiological or morphological phenotypes.

## Materials and methods

**Key resources table:** 

Reagent type (species) or resource	Designation	Source or reference	Identifiers	Additional information
Software, algorithm	CESAR	https://github.com/hillerlab/CESAR2.0		
Software, algorithm	Lastz (1.03.54)	http://www.bx.psu.edu/~rsharris/lastz/newer/lastz-1.03.54.tar.gz		K = 2400 L = 3000
Software, algorithm	axtChain	https://github.com/ucscGenomeBrowser/kent		
Software, algorithm	chainNet	https://github.com/ucscGenomeBrowser/kent		
Software, algorithm	BLAST (2.6.0+)	ftp://ftp.ncbi.nlm.nih.gov/blast/executables/blast+/LATEST/ncbi-blast-2.6.0+-x64-linux.tar.gz	RRID:SCR_004870	word_size = 7
Software, algorithm	MACSE (2.01)	https://bioweb.supagro.inra.fr/macse/		-prog enrichAlignment -prog refineAlignment
Software, algorithm	RELAX (HYPHY 2.3.11)	https://github.com/veg/hyphy	RRID:SCR_016162	
Software, algorithm	PAML (4.0)	http://abacus.gene.ucl.ac.uk/software/paml.html	RRID:SCR_014932	
Software, algorithm	TargetP (1.1)	http://www.cbs.dtu.dk/services/TargetP/		
Software, algorithm	HISAT2 (2.0.0)	https://ccb.jhu.edu/software/hisat2/index.shtml	RRID:SCR_015530	
Software, algorithm	SAMtools (1.1)	https://github.com/samtools/samtools	RRID:SCR_002105	

### Investigating the *HMGCS2* reading frame

To investigate the *HMGCS2* sequence across mammals, we used a whole genome alignment between the human reference genome (hg38 assembly) and 69 other placental mammal genomes (Sharma and Hiller, 2017). In addition to these assemblies, we downloaded the genome assembly of the Hippopotamus (Árnason et al., 2018) (NCBI GCA_002995585.1) and updated genome assemblies of the Large flying fox (NCBI GCF_000151845.1), the Egyptian fruit bat (NCBI GCF_001466805.2) and the African savanna elephant (ftp://ftp.broadinstitute.org/distribution/assemblies/mammals/elephant/loxAfr4/). For these four assemblies, we computed pairwise alignment chains to the human hg38 genome by applying lastz (Harris, 2007) with parameters K = 2400, L = 3000 and the default scoring matrix, axtChain (Kent et al., 2003) and chainCleaner (Suarez et al., 2017) (both with default parameters). Collinear alignment chains were visualized in the UCSC genome browser (Casper et al., 2018) and inspected for conserved synteny with adjacent genes. All analyzed genome assemblies are listed in Supplementary file 4.

We used the gene loss detection approach (Sharma et al., 2018a) to search across all mammals for mutations that could inactivate *HMGCS2*. This approach considers large deletions that cover exons, frameshifting insertions and deletions, mutations that disrupt donor (GT/GC) or acceptor (AG) splice site dinucleotides, and nonsense mutations. To exclude false inactivating mutations caused by alignment ambiguities, this method only considers those putative inactivating mutations that were confirmed by CESAR (Sharma et al., 2016; Sharma et al., 2017), a method trained to output an intact exon alignment whenever possible. Furthermore, exon deletions or exonic regions that do not align between human and another species were only considered if the respective locus did not overlap an assembly gap in the other genome (Hiller et al., 2012). For the proboscis monkey and lesser Egyptian jerboa, greater than 20% of the *HMGCS2* protein-coding region was ambiguous bases due to assembly gaps. These species were classified as ‘missing’, as it is not possible to unambiguously determine presence or absence of *HMGCS2*.

### Validation of gene-inactivating mutations

Exon losses and inactivating mutations identified were manually validated using unassembled sequencing read data from the TRACE and Sequence Read Archives. To validate exon losses, we used sensitive blastn runs (word size = 7) to search read data of *HMGCS2* loss species. As queries, we used *HMGSC1* and *HMGCS2* exon sequences from a closely related species with an intact *HMGCS2* gene. Specifically, we used the cow sequence to search cetacean read data, and the sequence of David’s myotis bat to search pteropodid read data. Read data from elephants and mastodon was searched using the manatee *HMGSC1*, *HMGCS2* and *REG4* exonic sequence. To validate smaller inactivating mutations (stop codon, frameshift and splice site mutations) and exon deletions, we extracted the genomic context 50 bp up- and downstream of each inactivating mutation in an *HMGCS2* loss species and determined the number of sequencing reads that support the derived (inactivating) and ancestral (non-inactivating) allele, as described in (Hecker et al., 2017). SRA accessions are provided in Supplementary file 5.

### Relaxed selection analysis

We generated a multiple sequence alignment of the *HMGCS2* coding sequence from the CESAR alignments and replaced in-frame stop codons with ‘NNN’. Using MACSE v2 (Ranwez et al., 2018), we added to this alignment the Chinese Horseshoe bat (*Rhinolophus sinicus*, XM_019730577) and the *Hippopotamus amphibius HMGCS2* coding sequence as well as the inferred exonic sequences of the Asian elephant, the African forest elephant and the mastodon. The alignment was then refined using MACSE v2 prior to visual inspection and further refinement. RELAX (Wertheim et al., 2015) was applied to test for relaxation of selection. First, we designated all branches within the cetacean, pteropodid and elephant/mastodon subtrees as foreground and designated all other branches as background. Second, we tested each subtree separately against the background branches, removing the other two *HMGCS2* loss lineages. We also tested the elephant lineage including only the African savanna elephant.

### Molecular dating of *HMGCS2* loss in the elephant lineage

To date the loss of *HMGCS2* along the putative loss branches in the phylogenetic tree, we used the method described in (Meredith et al., 2009; Gaudry et al., 2017), which estimates the portion of the loss branch where the gene evolved under selection and the portion where it evolved neutrally. Since synonymous positions do not entirely evolve neutrally due to constraints on splicing and translation, this approach assumes that the synonymous mutation rate of a functional gene is 70% of the fully-neutral synonymous mutation rate of an inactivated gene. Upper and lower bounds of species divergence times, the estimated length of the loss branch and respective sources are given in Supplementary file 3. The branch model in PAML (Yang, 2007) was fit, with five dN/dS classes, one for each of the three loss branches, one for the subsequent pseudogene branches and a final class for all functional branches. Pseudogene branches were assumed to evolve with a dN/dS of 1 for the dating calculations. We also fit models for each loss lineage individually and further tested the elephant lineage including only the African savanna elephant.

### Investigating the possibility of co-option of *HMGCS1*

We tested the amino acid sequences of the annotated or CESAR-inferred HMGCS1 protein from all *HMGCS2*-loss species for the presence of a potential mitochondrial target peptide (mTP) using TargetP (Emanuelsson et al., 2007). This revealed no evidence for the presence of an mTP in any species. To investigate the possibility that an mTP is provided by a novel or alternative first coding exon, we inspected gene predictions from Augustus that were available for all species. Those predicted gene models that contained an alternative first exon were found to not have an mTP. Furthermore, we used RNA-seq data from liver, the primary site of ketogenesis in other species, which was available for the Egyptian fruit bat (SRA SRR2914059, SRR2914369) and the minke whale (SRR919296). RNA-seq reads were mapped to the genome using HISAT2 (Kim et al., 2015), SAM files were sorted and converted to BAM files using SAMtools (Li et al., 2009) prior to visualization in the UCSC genome browser. For both species, we found no evidence of alternative or novel exons that could result in a different HMGCS1 N-terminus.

### Investigating the loss of other ketogenesis enzymes

Three other genes, *ACAT1*, *HMGCL* and *BDH1*, which encode components of the ketogenesis pathway were investigated for potential inactivating mutations using the same gene loss pipeline and mutation validation strategy described above. These genes were also tested for signs of relaxed selection in the three *HMGCS2-*loss lineages using RELAX (Supplementary file 2).

### Data availability

All data analyzed during this study is publicly available on NCBI, SRA and the Trace Archive. The multiple sequence alignment of the mammalian *HMGCS2* coding sequences is provided as Figure 2—source data 1.

## Additional files

Supplementary file 1. Number of unassembled sequence reads supporting gene-inactivating mutations in cetaceans and fruit bats. Please note that sequencing errors can change a real stop codon to a sense codon, which happened in two cases; however, given that > 60 reads confirm the stop codon, these single erroneous reads to not support the presence of the ancestral allele. The heterozygous 1 bp deletion in the black flying fox (last row) is discussed in the main text.

Supplementary file 2. RELAX analysis of genes in the ketogenesis pathway. The estimate for ‘All’ and Elephantimorpha was computed from an alignment including the Asian elephant, the African forest elephant and the mastodon.

Supplementary file 3. Dating the loss of *HMGCS2*. The table lists divergence times and estimates for how long *HMGCS2* was functional along the mixed branch leading to the three loss lineages. Using the method of (Meredith et al., 2009; Gaudry et al., 2017), we calculated a point estimate for when *HMGCS2* was lost and an upper/lower bound of this estimate. (A) Elephant lineage. We estimated *HMGCS2* loss dates both from an alignment that includes sequences of Elephantimorpha and from an alignment that includes only the African savanna elephant. (B) Fruit bat lineage. (C) Cetacean lineage.

Supplementary file 4. Species and genome assemblies that were analyzed in this study.

Supplementary file 5. Sources of unassembled genomic sequencing reads used for the validation of inactivating mutations in *HMGCS2* loss species

Transparent reporting form

### Data availability

All data analyzed during this study are publicly available on NCBI, SRA and the Trace Archive. The alignment used to date gene loss provided as a source file.

The following previously published datasets were used:

Zhang GCowled CShi ZHuang ZBishop-Lilly KAFang XWynne JWXiong ZBaker MLZhao WTachedjian MZhu YZhou PJiang XNg JYang LWu LXiao JFeng Y2013Black flying fox raw sequence readshttps://www.ncbi.nlm.nih.gov/sra/SRX174444Publicly available at the NCBI Sequence Read Archive (accession no. SRX174444)

Zhang GCowled CShi ZHuang ZBishop-Lilly KAFang XWynne JWXiong ZBaker MLZhao WTachedjian MZhu YZhou PJiang XNg JYang LWu LXiao JFeng YChen YSun XZhang YMarsh GACrameri GBroder CCFrey KGWang LFWang J.2013Black flying fox raw sequence readshttps://www.ncbi.nlm.nih.gov/sra/SRX174445Publicly available at the NCBI Sequence Read Archive (accession no. SRX174445)

Zhang GCowled CShi ZHuang ZBishop-Lilly KAFang XWynne JWXiong ZBaker MLZhao WTachedjian MZhu YZhou PJiang XNg JYang LWu LXiao JFeng YChen YSun XZhang YMarsh GACrameri GBroder CCFrey KGWang LFWang J.2013Black flying fox raw sequence readshttps://www.ncbi.nlm.nih.gov/sra/SRX174446Publicly available at the NCBI Sequence Read Archive (accession no. SRX174446

Zhang GCowled CShi ZHuang ZBishop-Lilly KAFang XWynne JWXiong ZBaker MLZhao WTachedjian MZhu YZhou PJiang XNg JYang LWu LXiao JFeng YChen YSun XZhang YMarsh GACrameri GBroder CCFrey KGWang LFWang J.2013Black flying fox raw sequence readshttps://www.ncbi.nlm.nih.gov/sra/SRX174448Publicly available at the NCBI Sequence Read Archive (accession no. SRX174448)

Kerstin Lindblad-TohManuel GarberOr ZukMichael F. LinBrian J. ParkerStefan WashietlPouya KheradpourJason ErnstGregory JordanEvan MauceliLucas D. WardCraig B. LoweAlisha K. HollowayMichele ClampSante GnerreJessica AlföldiKathryn BealJean ChangHiram ClawsonJames CuffFederica Di PalmaStephen FitzgeraldPaul FlicekMitchell GuttmanMelissa J. HubiszDavid B. JaffeIrwin JungreisW. James KentDennis KostkaMarcia LaraAndre L. MartinsTim MassinghamIda MoltkeBrian J. RaneyMatthew D. RasmussenJim RobinsonAlexander StarkAlbert J. VilellaJiayu WenXiaohui XieMichael C. ZodyBroad Institute Sequencing Platform and Whole Genome Assembly TeamKim C. WorleyChristie L. KovarDonna M. MuznyRichard A. GibbsBaylor College of Medicine Human Genome Sequencing Center Sequencing TeamWesley C. WarrenElaine R. MardisGeorge M. WeinstockRichard K. WilsonGenome Institute at Washington UniversityEwan BirneyElliott H. MarguliesJavier HerreroEric D. GreenDavid HausslerAdam SiepelNick GoldmanKatherine S. PollardJakob S. PedersenEric S. Lander & Manolis Kellis2011Large flying fox raw sequence readshttps://www.hgsc.bcm.edu/other-mammals/megabat-genome-projectPublicly available at the Human Genome Sequencing Center

Kerstin Lindblad-TohManuel GarberOr ZukMichael F. LinBrian J. ParkerStefan WashietlPouya KheradpourJason ErnstGregory JordanEvan MauceliLucas D. WardCraig B. LoweAlisha K. HollowayMichele ClampSante GnerreJessica AlföldiKathryn BealJean ChangHiram ClawsonJames CuffFederica Di PalmaStephen FitzgeraldPaul FlicekMitchell GuttmanMelissa J. HubiszDavid B. JaffeIrwin JungreisW. James KentDennis KostkaMarcia LaraAndre L. MartinsTim MassinghamIda MoltkeBrian J. RaneyMatthew D. RasmussenJim RobinsonAlexander StarkAlbert J. VilellaJiayu WenXiaohui XieMichael C. ZodyBroad Institute Sequencing Platform and Whole Genome Assembly TeamKim C. WorleyChristie L. KovarDonna M. MuznyRichard A. GibbsBaylor College of Medicine Human Genome Sequencing Center Sequencing TeamWesley C. WarrenElaine R. MardisGeorge M. WeinstockRichard K. WilsonGenome Institute at Washington UniversityEwan BirneyElliott H. MarguliesJavier HerreroEric D. GreenDavid HausslerAdam SiepelNick GoldmanKatherine S. PollardJakob S. PedersenEric S. Lander & Manolis Kellis2011Large flying fox raw sequence readshttps://www.ncbi.nlm.nih.gov/sra/SRX708163Publicly available at the NCBI Sequence Read Archive (accession no. SRX708163)

Kerstin Lindblad-TohManuel GarberOr ZukMichael F. LinBrian J. ParkerStefan WashietlPouya KheradpourJason ErnstGregory JordanEvan MauceliLucas D. WardCraig B. LoweAlisha K. HollowayMichele ClampSante GnerreJessica AlföldiKathryn BealJean ChangHiram ClawsonJames CuffFederica Di PalmaStephen FitzgeraldPaul FlicekMitchell GuttmanMelissa J. HubiszDavid B. JaffeIrwin JungreisW. James KentDennis KostkaMarcia LaraAndre L. MartinsTim MassinghamIda MoltkeBrian J. RaneyMatthew D. RasmussenJim RobinsonAlexander StarkAlbert J. VilellaJiayu WenXiaohui XieMichael C. ZodyBroad Institute Sequencing Platform and Whole Genome Assembly TeamKim C. WorleyChristie L. KovarDonna M. MuznyRichard A. GibbsBaylor College of Medicine Human Genome Sequencing Center Sequencing TeamWesley C. WarrenElaine R. MardisGeorge M. WeinstockRichard K. WilsonGenome Institute at Washington UniversityEwan BirneyElliott H. MarguliesJavier HerreroEric D. GreenDavid HausslerAdam SiepelNick GoldmanKatherine S. PollardJakob S. PedersenEric S. Lander & Manolis Kellis2011Large flying fox raw sequence readshttps://www.ncbi.nlm.nih.gov/sra/SRX708167Publicly available at the NCBI Sequence Read Archive (accession no. SRX708167)

Kerstin Lindblad-TohManuel GarberOr ZukMichael F. LinBrian J. ParkerStefan WashietlPouya KheradpourJason ErnstGregory JordanEvan MauceliLucas D. WardCraig B. LoweAlisha K. HollowayMichele ClampSante GnerreJessica AlföldiKathryn BealJean ChangHiram ClawsonJames CuffFederica Di PalmaStephen FitzgeraldPaul FlicekMitchell GuttmanMelissa J. HubiszDavid B. JaffeIrwin JungreisW. James KentDennis KostkaMarcia LaraAndre L. MartinsTim MassinghamIda MoltkeBrian J. RaneyMatthew D. RasmussenJim RobinsonAlexander StarkAlbert J. VilellaJiayu WenXiaohui XieMichael C. ZodyBroad Institute Sequencing Platform and Whole Genome Assembly TeamKim C. WorleyChristie L. KovarDonna M. MuznyRichard A. GibbsBaylor College of Medicine Human Genome Sequencing Center Sequencing TeamWesley C. WarrenElaine R. MardisGeorge M. WeinstockRichard K. WilsonGenome Institute at Washington UniversityEwan BirneyElliott H. MarguliesJavier HerreroEric D. GreenDavid HausslerAdam SiepelNick GoldmanKatherine S. PollardJakob S. PedersenEric S. Lander & Manolis Kellis2011Large flying fox raw sequence readshttps://www.ncbi.nlm.nih.gov/sra/SRX708168Publicly available at the NCBI Sequence Read archive (accession no. SRX708168)

Kerstin Lindblad-TohManuel GarberOr ZukMichael F. LinBrian J. ParkerStefan WashietlPouya KheradpourJason ErnstGregory JordanEvan MauceliLucas D. WardCraig B. LoweAlisha K. HollowayMichele ClampSante GnerreJessica AlföldiKathryn BealJean ChangHiram ClawsonJames CuffFederica Di PalmaStephen FitzgeraldPaul FlicekMitchell GuttmanMelissa J. HubiszDavid B. JaffeIrwin JungreisW. James KentDennis KostkaMarcia LaraAndre L. MartinsTim MassinghamIda MoltkeBrian J. RaneyMatthew D. RasmussenJim RobinsonAlexander StarkAlbert J. VilellaJiayu WenXiaohui XieMichael C. ZodyBroad Institute Sequencing Platform and Whole Genome Assembly TeamKim C. WorleyChristie L. KovarDonna M. MuznyRichard A. GibbsBaylor College of Medicine Human Genome Sequencing Center Sequencing TeamWesley C. WarrenElaine R. MardisGeorge M. WeinstockRichard K. WilsonGenome Institute at Washington UniversityEwan BirneyElliott H. MarguliesJavier HerreroEric D. GreenDavid HausslerAdam SiepelNick GoldmanKatherine S. PollardJakob S. PedersenEric S. Lander & Manolis Kellis2011Large flying fox raw sequence readshttps://www.ncbi.nlm.nih.gov/sra/SRX708169Publicly available at the NCBI Sequence Read archive (accession no. SRX708169)

Beijing Genomics Institute2015Dolphin raw sequence readshttps://www.ncbi.nlm.nih.gov/sra/SRX1136398Publicly available at the NCBI Sequence Read Archive (accession no. SRX1136398)

Beijing Genomics Institute2015Dolphin raw sequence readshttps://www.ncbi.nlm.nih.gov/sra/SRX1136399Publicly available at the NCBI Sequence Read Archive (accession no. SRX1136399)

Beijing Genomics Institute2015Dolphin raw sequence readshttps://www.ncbi.nlm.nih.gov/sra/SRX1136400Publicly available at the NCBI Sequence Read Archive (accession no. SRX1136400)

The National Institute of Standards and Technology2016Dolphin raw sequence readshttps://www.ncbi.nlm.nih.gov/sra/SRX2439826Publicly available at the NCBI Sequence Read Archive (accession no. SRX2439826)

The National Institute of Standards and Technology2016Dolphin raw sequence readshttps://www.ncbi.nlm.nih.gov/sra/SRX2439828Publicly available at the NCBI Sequence Read Archive (accession no. SRX2439828)

The National Institute of Standards and Technology2016Dolphin raw sequence readshttps://www.ncbi.nlm.nih.gov/sra/SRX2439829Publicly available at the NCBI Sequence Read Archive (accession no. SRX2439829)

BirneyElliott H. MarguliesJavier HerreroEric D. GreenDavid HausslerAdam SiepelNick GoldmanKatherine S. PollardJakob S. PedersenEric S. Lander & Manolis Kellis2011Dolphin raw sequence readsftp://ftp-private.ncbi.nlm.nih.gov/pub/TraceDB/tursiops_truncatusAvailable to download from the NCBI FTP site

Foote ADLiu YThomas GWVinař TAlföldi JDeng JDugan Svan Elk CEHunter MEJoshi VKhan ZKovar CLee SLLindblad-Toh KMancia ANielsen RQin XQu JRaney BJVijay NWolf JBHahn MWMuzny DMWorley KCGilbert MTGibbs RA.2015Killer whale raw sequence readshttps://www.ncbi.nlm.nih.gov/sra/SRX188930Publicly available at the NCBI Sequence Read Archive (accession no. SRX188930)

Foote ADLiu YThomas GWVinař TAlföldi JDeng JDugan Svan Elk CEHunter MEJoshi VKhan ZKovar CLee SLLindblad-Toh KMancia ANielsen RQin XQu JRaney BJVijay NWolf JBHahn MWMuzny DMWorley KCGilbert MTGibbs RA.2015Killer whale raw sequence readshttps://www.ncbi.nlm.nih.gov/sra/SRX188933Publicly available at the NCBI Sequence Read Archive (accession no. SRX188933)

Hyung-Soon YimYun Sung ChoXuanmin GuangSung Gyun KangJae-Yeon JeongSun-Shin ChaHyun-Myung OhJae-Hak LeeEun Chan YangKae Kyoung KwonYun Jae KimTae Wan KimWonduck KimJeong Ho JeonSang-Jin KimDong Han ChoiSungwoong JhoHak-Min KimJunsu KoHyunmin KimYoung-Ah ShinHyun-Ju JungYuan ZhengZhuo WangYan ChenMing ChenAwei JiangErli LiShu ZhangHaolong HouTae Hyung KimLili YuSha LiuKung AhnJesse CooperSin-Gi ParkChang Pyo HongWook JinHeui-Soo KimChankyu ParkKyooyeol LeeSung ChunPhillip A MorinStephen J O'BrienHang LeeJumpei KimuraDae Yeon MoonAndrea ManicaJeremy EdwardsByung Chul KimSangsoo KimJun WangJong BhakHyun Sook Lee & Jung-Hyun Lee2014Minke whale raw sequence readshttps://www.ncbi.nlm.nih.gov/sra/SRX302112Publicly available at the NCBI Sequence Read Archive (accession no. SRX302112)

Hyung-Soon YimYun Sung ChoXuanmin GuangSung Gyun KangJae-Yeon JeongSun-Shin ChaHyun-Myung OhJae-Hak LeeEun Chan YangKae Kyoung KwonYun Jae KimTae Wan KimWonduck KimJeong Ho JeonSang-Jin KimDong Han ChoiSungwoong JhoHak-Min KimJunsu KoHyunmin KimYoung-Ah ShinHyun-Ju JungYuan ZhengZhuo WangYan ChenMing ChenAwei JiangErli LiShu ZhangHaolong HouTae Hyung KimLili YuSha LiuKung AhnJesse CooperSin-Gi ParkChang Pyo HongWook JinHeui-Soo KimChankyu ParkKyooyeol LeeSung ChunPhillip A MorinStephen J O'BrienHang LeeJumpei KimuraDae Yeon MoonAndrea ManicaJeremy EdwardsByung Chul KimSangsoo KimJun WangJong BhakHyun Sook Lee & Jung-Hyun Lee2015Minke whale raw sequence readshttps://www.ncbi.nlm.nih.gov/sra/SRX316745Publicly available at the NCBI Sequence Read Archive (accession no. SRX316745)

Ketil MaldeBjørghild B. SeliussenMaría QuintelaGeir DahleFrancois BesnierHans J. SkaugNils ØienHiroko K. SolvangTore HaugRasmus Skern-MauritzenNaohisa KandaLuis A. PasteneInge Jonassen and Kevin A. Glover2016Minke whale raw sequence readshttps://www.ncbi.nlm.nih.gov/sra/SRX2007381Publicly available at the NCBI Sequence Read Archive (accession no. SRX2007381)

Ketil MaldeBjørghild B. SeliussenMaría QuintelaGeir DahleFrancois BesnierHans J. SkaugNils ØienHiroko K. SolvangTore HaugRasmus Skern-MauritzenNaohisa KandaLuis A. PasteneInge Jonassen and Kevin A. Glover2016Minke whale sequence readshttps://www.ncbi.nlm.nih.gov/sra/SRX2007379Publicly available at the NCBI Sequence Read Archive (accession no. SRX2007379)

Ketil MaldeBjørghild B. SeliussenMaría QuintelaGeir DahleFrancois BesnierHans J. SkaugNils ØienHiroko K. SolvangTore HaugRasmus Skern-MauritzenNaohisa KandaLuis A. PasteneInge Jonassen and Kevin A. Glover2016Minke whale sequence reads archivehttps://www.ncbi.nlm.nih.gov/sra/SRX2007373Publicly available at the NCBI Read Archive (accession no. SRX2007373)

Ketil MaldeBjørghild B. SeliussenMaría QuintelaGeir DahleFrancois BesnierHans J. SkaugNils ØienHiroko K. SolvangTore HaugRasmus Skern-MauritzenNaohisa KandaLuis A. PasteneInge Jonassen and Kevin A. Glover\2016Minke whale sequence readshttps://www.ncbi.nlm.nih.gov/sra/SRX2007368Publicly available at the NCBI Sequence Read Archive (accession no. SRX2007368)

Wesley C. WarrenLukas KudernaAlana AlexanderJulian CatchenJose G. Perez-SilvaCarlos Lopez-OtınVıctor QuesadaPatrick MinxChad TomlinsonMichael J. MontagueFabiana H. G. FariasRonald B. WalterTomas Marques-BonetTravis GlennTroy J. KieranSandra S. WiseJohn Pierce Wise JrRobert M. Waterhouseand John Pierce Wise Sr2017Sperm whale sequence readshttps://www.ncbi.nlm.nih.gov/sra/SRX220365Publicly available at the NCBI Sequence Read Archive (accession no. SRX220365)

Wesley C. WarrenLukas KudernaAlana AlexanderJulian CatchenJose G. Perez-SilvaCarlos Lopez-OtınVıctor QuesadaPatrick MinxChad TomlinsonMichael J. MontagueFabiana H. G. FariasRonald B. WalterTomas Marques-BonetTravis GlennTroy J. KieranSandra S. WiseJohn Pierce Wise JrRobert M. Waterhouseand John Pierce Wise Sr2017Sperm whale raw sequence readshttps://www.ncbi.nlm.nih.gov/sra/SRX220366Publicly available at the NCBI Sequence Read Archive (accession no. SRX220366)

Wesley C. WarrenLukas KudernaAlana AlexanderJulian CatchenJose G. Perez-SilvaCarlos Lopez-OtınVıctor QuesadaPatrick MinxChad TomlinsonMichael J. MontagueFabiana H. G. FariasRonald B. WalterTomas Marques-BonetTravis GlennTroy J. KieranSandra S. WiseJohn Pierce Wise JrRobert M. Waterhouseand John Pierce Wise Sr2017Sperm whale sequence readshttps://www.ncbi.nlm.nih.gov/sra/SRX220367Publicly available at the NCBI Sequence Read Archive (accession no. SRX220367)

Puli Chandramouli ReddyIshani SinhaAshwin KelkarFarhat HabibSaurabh J. PradhanRaman SukumarSanjeev Galande2015Asian elephant raw sequence readshttps://www.ncbi.nlm.nih.gov/sra/SRX1427123Publicly available at the NCBI Sequence Read Archive (accession no. SRX1427123)

Palkopoulou ELipson MMallick SNielsen SRohland NBaleka SKarpinski EIvancevic AMTo THKortschak RDRaison JMQu ZChin TJAlt KWClaesson SDalén LMacPhee RDEMeller HRoca ALRyder OAHeiman DYoung SBreen MWilliams CAken BLRuffier MKarlsson EJohnson JDi Palma FAlfoldi JAdelson DLMailund TMunch KLindblad-Toh KHofreiter MPoinar HReich D2018Forest elephant raw sequence readshttps://www.ebi.ac.uk/ena/data/view/ERR2260500Publicly available at the NCBI Sequence Read Archive (accession no. ERR2260500)

Palkopoulou ELipson MMallick SNielsen SRohland NBaleka SKarpinski EIvancevic AMTo THKortschak RDRaison JMQu ZChin TJAlt KWClaesson SDalén LMacPhee RDEMeller HRoca ALRyder OAHeiman DYoung SBreen MWilliams CAken BLRuffier MKarlsson EJohnson JDi Palma FAlfoldi JAdelson DLMailund TMunch KLindblad-Toh KHofreiter MPoinar HReich D2018Forest elephant raw sequence readshttps://www.ebi.ac.uk/ena/data/view/ERR2260495Publicly available at the European Nucleotide Archive (accession no. ERR2260495)

Diego CortezRay MarinDeborah Toledo-FloresLaure FroidevauxAngélica LiechtiPaul D. WatersFrank Grützner & Henrik Kaessmann2014Savanna elephant raw sequence readshttps://www.ncbi.nlm.nih.gov/sra/SRX339471Publicly available at the NCBI Sequence Read Archive (accession no. SRX339471)

Diego CortezRay MarinDeborah Toledo-FloresLaure FroidevauxAngélica LiechtiPaul D. WatersFrank Grützner & Henrik Kaessmann2014Savanna elephant raw sequence readshttps://www.ncbi.nlm.nih.gov/sra/SRX339470Publicly available at the NCBI Sequence Read Archive (accession no. SRX339470)

Palkopoulou ELipson MMallick SNielsen SRohland NBaleka SKarpinski EIvancevic AMTo THKortschak RDRaison JMQu ZChin TJAlt KWClaesson SDalén LMacPhee RDEMeller HRoca ALRyder OAHeiman DYoung SBreen MWilliams CAken BLRuffier MKarlsson EJohnson JDi Palma FAlfoldi JAdelson DLMailund TMunch KLindblad-Toh KHofreiter MPoinar HReich D2018American mastodon raw sequence readshttps://www.ebi.ac.uk/ena/data/view/ERR2260508Publicly available at the European Nucleotide Archive (accession no.ERR2260508)

Palkopoulou ELipson MMallick SNielsen SRohland NBaleka SKarpinski EIvancevic AMTo THKortschak RDRaison JMQu ZChin TJAlt KWClaesson SDalén LMacPhee RDEMeller HRoca ALRyder OAHeiman DYoung SBreen MWilliams CAken BLRuffier MKarlsson EJohnson JDi Palma FAlfoldi JAdelson DLMailund TMunch KLindblad-Toh KHofreiter MPoinar HReich D2018American mastodon raw sequence readshttps://www.ebi.ac.uk/ena/data/view/ERR2260503Publicly available at the European Nucleotide Archive (accession no. ERR2260503)

Hyung-Soon YimYun Sung ChoXuanmin GuangSung Gyun KangJae-Yeon JeongSun-Shin ChaHyun-Myung OhJae-Hak LeeEun Chan YangKae Kyoung KwonYun Jae KimTae Wan KimWonduck KimJeong Ho JeonSang-Jin KimDong Han ChoiSungwoong JhoHak-Min KimJunsu KoHyunmin KimYoung-Ah ShinHyun-Ju JungYuan ZhengZhuo WangYan ChenMing ChenAwei JiangErli LiShu ZhangHaolong HouTae Hyung KimLili YuSha LiuKung AhnJesse CooperSin-Gi ParkChang Pyo HongWook JinHeui-Soo KimChankyu ParkKyooyeol LeeSung ChunPhillip A MorinStephen J O'BrienHang LeeJumpei KimuraDae Yeon MoonAndrea ManicaJeremy EdwardsByung Chul KimSangsoo KimJun WangJong BhakHyun Sook Lee & Jung-Hyun Lee2016Minke whale raw sequence readshttps://www.ncbi.nlm.nih.gov/sra/SRX318063Publicly available at the Sequence Read Archive (accession no. SRX318063)
