# Supplementary material for: Recurrent loss of HMGCS2 shows that ketogenesis is not essential for the evolution of large mammalian brains
Source: eLife. 2018 Oct 16;7:e38906. doi: 10.7554/eLife.38906 (PMC6191284; doi:10.7554/eLife.38906)
Supplement: Transparent reporting form [file elife-38906-transrepform.docx]

***eLife’s* transparent reporting form**

We encourage authors to provide detailed information *within their submission* to facilitate the interpretation and replication of experiments. Authors can upload supporting documentation to indicate the use of appropriate reporting guidelines for health-related research (see [EQUATOR Network](http://www.equator-network.org/%20)), life science research (see the [BioSharing Information Resource](https://biosharing.org/)), or the [ARRIVE guidelines](http://www.plosbiology.org/article/info:doi/10.1371/journal.pbio.1000412) for reporting work involving animal research. Where applicable, authors should refer to any relevant reporting standards documents in this form.

If you have any questions, please consult our Journal Policies and/or contact us: [editorial@elifesciences.org](mailto:editorial@elifesciences.org).

**Sample-size estimation**

- You should state whether an appropriate sample size was computed when the study was being designed
- You should state the statistical method of sample size computation and any required assumptions
- If no explicit power analysis was used, you should describe how you decided what sample (replicate) size (number) to use

Please outline where this information can be found within the submission (e.g., sections or figure legends), or explain why this information doesn’t apply to your submission:

We used publicly available genomic sequence data, to compare representative genomes across species. No sample size computation or power analysis was necessary for this study.

**Replicates**

- You should report how often each experiment was performed
- You should include a definition of biological versus technical replication
- The data obtained should be provided and sufficient information should be provided to indicate the number of independent biological and/or technical replicates
- If you encountered any outliers, you should describe how these were handled
- Criteria for exclusion/inclusion of data should be clearly stated
- High-throughput sequence data should be uploaded before submission, with a private link for reviewers provided (these are available from both GEO and ArrayExpress)

Please outline where this information can be found within the submission (e.g., sections or figure legends), or explain why this information doesn’t apply to your submission:

As this is a comparative genomics study using all available data, no replicates were performed.

**Statistical reporting**

- Statistical analysis methods should be described and justified
- Raw data should be presented in figures whenever informative to do so (typically when N per group is less than 10)
- For each experiment, you should identify the statistical tests used, exact values of N, definitions of center, methods of multiple test correction, and dispersion and precision measures (e.g., mean, median, SD, SEM, confidence intervals; and, for the major substantive results, a measure of effect size (e.g., Pearson's r, Cohen's d)
- Report exact p-values wherever possible alongside the summary statistics and 95% confidence intervals. These should be reported for all key questions and not only when the p-value is less than 0.05.

Please outline where this information can be found within the submission (e.g., sections or figure legends), or explain why this information doesn’t apply to your submission:

Phylogenetic hypothesis testing was performed using the RELAX module from the HYPHY package. P-values are reported for each test in Supplementary Table 2.

(For large datasets, or papers with a very large number of statistical tests, you may upload a single table file with tests, Ns, etc., with reference to sections in the manuscript.)

**Group allocation**

- Indicate how samples were allocated into experimental groups (in the case of clinical studies, please specify allocation to treatment method); if randomization was used, please also state if restricted randomization was applied
- Indicate if masking was used during group allocation, data collection and/or data analysis

Please outline where this information can be found within the submission (e.g., sections or figure legends), or explain why this information doesn’t apply to your submission:

Species were allocated as “loss” species if they possessed inactivating mutations in the coding domain of the *HMGCS2* gene. Species for which greater than 20% of the CDS was covered by assembly gaps were excluded from the analysis, as described in Methods-Investigating the HMGCS2 reading frame.

**Additional data files (“source data”)**

- We encourage you to upload relevant additional data files, such as numerical data that are represented as a graph in a figure, or as a summary table
- Where provided, these should be in the most useful format, and they can be uploaded as “Source data” files linked to a main figure or table
- Include model definition files including the full list of parameters used
- Include code used for data analysis (e.g., R, MatLab)
- Avoid stating that data files are “available upon request”

Please indicate the figures or tables for which source data files have been provided:

Alignment data used in this study is already publicly available from the UCSC or previously published genome alignments of Sharma and Hiller, NAR 2017.

The identifiers for raw sequence data used for validation of inactivating mutations are given in Supplementary Table 5. Identifiers of publicly-available RNA-seq data are included in the methods. Divergence time estimates used for molecular dating of *HMGCS2* loss in elephants and their source are given in Supplementary Table 3.

The sequence alignment that was used to estimate divergence times is provided in Figure 2-source data.
